# Pathological Predictors of Shunt Stenosis and Hepatic Encephalopathy after Transjugular Intrahepatic Portosystemic Shunt

**DOI:** 10.1155/2016/3681731

**Published:** 2016-11-15

**Authors:** Fuliang He, Shan Dai, Zhibo Xiao, Lei Wang, Zhendong Yue, Hongwei Zhao, Mengfei Zhao, Qiushi Lin, Xiaoqun Dong, Fuquan Liu

**Affiliations:** ^1^Department of Interventional Therapy, Beijing Shijitan Hospital, Capital Medical University, The 9th Affiliated Hospital of Peking University, Beijing 100038, China; ^2^Department of Plastic Surgery, The Second Affiliated Hospital of Harbin Medical University, Harbin 150081, China; ^3^Department of Internal Medicine, Section of Hematology-Oncology, Section of Gastroenterology, Stephenson Cancer Center, College of Medicine, University of Oklahoma Health Sciences Center, Oklahoma City, OK 73104, USA

## Abstract

*Background.* Transjugular intrahepatic portosystemic shunt (TIPS) is an artificial channel from the portal vein to the hepatic vein or vena cava for controlling portal vein hypertension. The major drawbacks of TIPS are shunt stenosis and hepatic encephalopathy (HE); previous studies showed that post-TIPS shunt stenosis and HE might be correlated with the pathological features of the liver tissues. Therefore, we analyzed the pathological predictors for clinical outcome, to determine the risk factors for shunt stenosis and HE after TIPS.* Methods.* We recruited 361 patients who suffered from portal hypertension symptoms and were treated with TIPS from January 2009 to December 2012.* Results.* Multivariate logistic regression analysis showed that the risk of shunt stenosis was increased with more severe inflammation in the liver tissue (OR, 2.864; 95% CI: 1.466–5.592; *P* = 0.002), HE comorbidity (OR, 6.266; 95% CI, 3.141–12.501; *P* < 0.001), or higher MELD score (95% CI, 1.298–1.731; *P* < 0.001). Higher risk of HE was associated with shunt stenosis comorbidity (OR, 6.266; 95% CI, 3.141–12.501; *P* < 0.001), higher stage of the liver fibrosis (OR, 2.431; 95% CI, 1.355–4.359; *P* = 0.003), and higher MELD score (95% CI, 1.711–2.406; *P* < 0.001).* Conclusion.* The pathological features can predict individual susceptibility to shunt stenosis and HE.

## 1. Introduction

Portal hypertension is defined as an increase in the blood pressure of portal venous system [1]. Portal vein pressure ranges between 1 and 4 mmHg higher than the hepatic vein (HV) pressure and not more than 6 mmHg higher than right atrial pressure [2]. Portal hypertension is defined as portal pressures that exceed these limits. Transjugular intrahepatic portosystemic shunt (TIPS) is an artificial channel from the portal vein to the hepatic vein. TIPS has been demonstrated as an effective procedure to control serious complications including gastrointestinal bleeding and refractory ascites in patients with portal hypertension caused by liver cirrhosis, Budd-Chiari syndrome (BCS), and other liver diseases. The technical success rate of TIPS has reached 95–100% whereas an operation-related mortality rate was only 1%. It can manage >90% gastrointestinal bleeding and 50–92% refractory ascites [3]. It is thus well accepted that TIPS plays an important role in treatment of patients with portal hypertension syndrome.

The major drawbacks of TIPS are shunt stenosis and hepatic encephalopathy (HE), which dramatically reduce the prognosis of TIPS and influence the patients' quality of life [4]. Our previous study showed that post-TIPS HE which occurred within 3 months was associated with high MELD score [5]. Several recent studies have revealed that the incidence of shunt stenosis and HE might be correlated with the pathological features of the liver tissues [6, 7]. Therefore, it is of great interest to elucidate whether pathological disorders of cirrhosis liver tissue confer the risk for post-TIPS shunt stenosis and HE. Thus, we performed biopsy of the liver tissues from the original shunt to each patient during TIPS in our single center. We analyzed the clinical and pathological data of the patients enrolled to determine the risk factors for HE and shunt stenosis after TIPS.

## 2. Materials and Methods

### 2.1. Clinical Data of the Patients

This study was approved by Institutional Review Board (IRB) committee at Beijing Shijitan Hospital. All procedures were carried out according to the guidelines approved by the ethics committee at Beijing Shijitan Hospital (approval number: 2008001). Informed consents of the procedures and data collection were acquired from all the patients and their families.

Inclusion and exclusion criteria were carefully designed to exclude the confounding factors. The inclusion criteria were (1) portal hypertension caused by hepatitis B-related cirrhosis; (2) indications for TIPS treatment: secondary prevention for variceal bleeding and/or refractory ascites; (3) signed informed consent; and (4) aged between 18 and 75 years. The patients with one or more of the following characteristics were excluded: (1) patients with portal hypertension combined with primary or metastatic liver tumors, (2) combined with HE before the treatment, (3) combined with active variceal bleeding (the time frame of the acute bleeding episode should be 3 days), and (4) combined with hemorrhage of gastrointestinal ulcer; (5) patients with history of TIPS placement or shunt surgery; (6) patients with severe cardiopulmonary diseases; and (7) patients with uncontrolled systemic infection.

**Figure 1:**
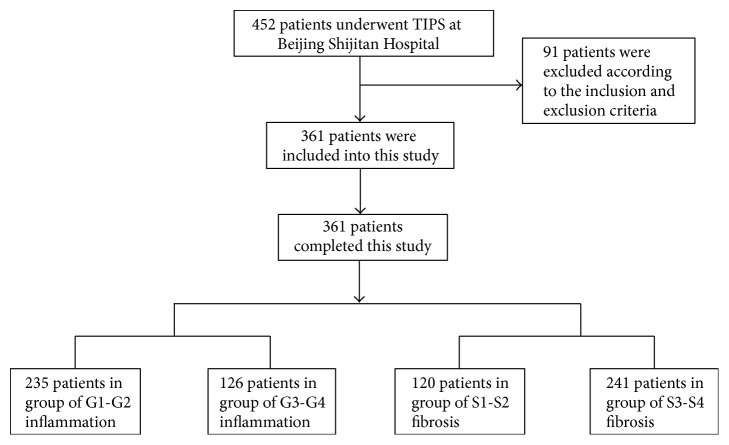
Enrollment flowchart of patients included in this study.

**Figure 2:**
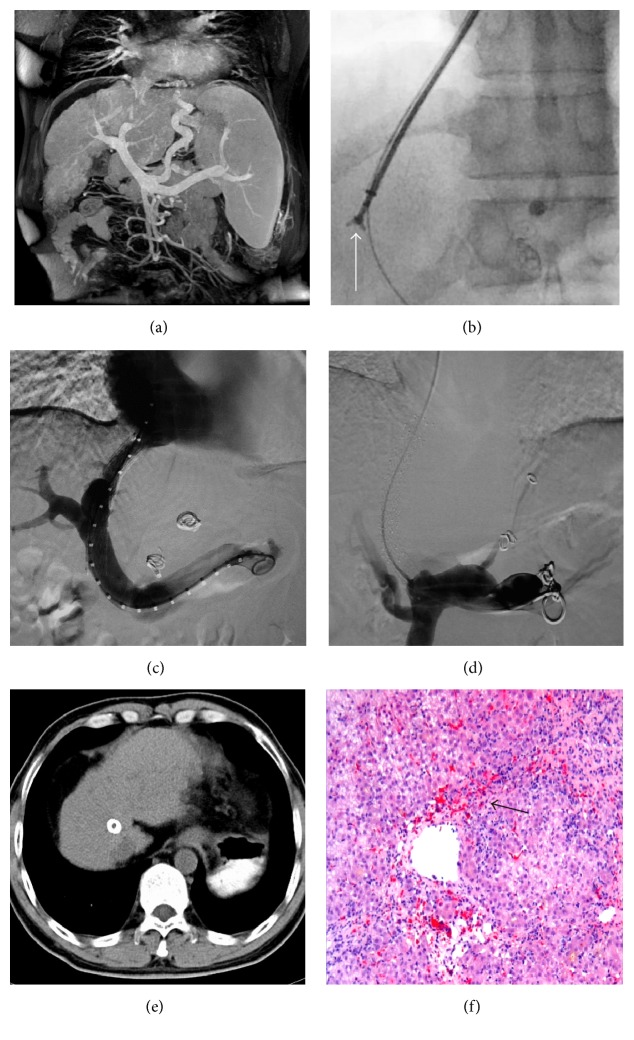
A representative case of shunt restenosis. This was from a 42 y/o male patient, who was diagnosed with hepatitis B-related liver cirrhosis and had suffered from gastrointestinal bleeding 2 weeks before hospitalization. (a) Coronal image of MRPV showed severe cirrhosis and portal hypertension leading to gastric coronary vein varices and splenomegaly. (b) Biopsy with the forceps before balloon dilation of the shunt under X-ray (the arrow pointed at the tip of the forceps). (c) Angiography showed that TIPS was performed successfully after the biopsy, with no procedure-related complications. (d) Angiography after 16 months of TIPS showed that the shunt was totally occluded. (e) The transverse image of CT after 16 months of TIPS highlighted the positon of the stent and revealed that the cirrhosis was still severe. (f) Pathological diagnosis demonstrated the widespread intralobular bridging necrosis with multiple hepatic lobules involved (arrow), which meant stage III inflammation (H&E staining, ×100).

**Figure 3:**
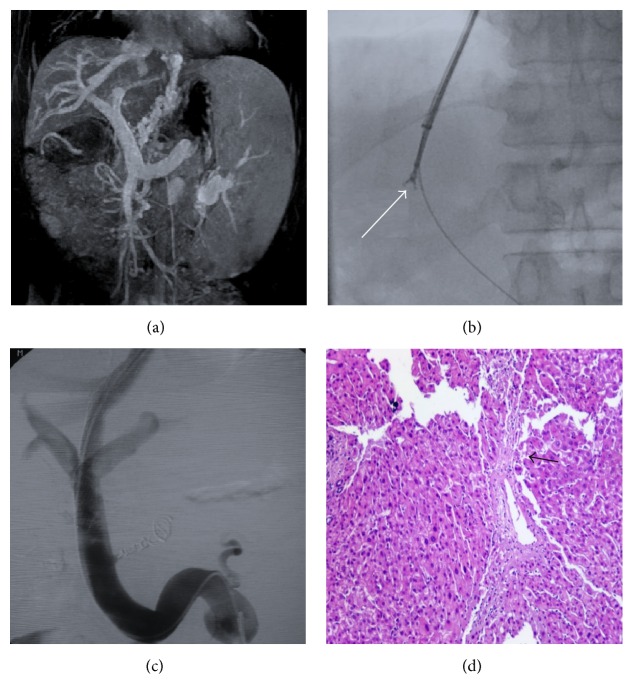
A representative case of HE after TIPS. This was from a 56 y/o male patient diagnosed with hepatitis B-related cirrhosis, who had suffered from gastrointestinal bleeding 4 weeks before hospitalization. The patient developed HE at 1 month after TIPS. (a) Coronal image of MRPV showed liver cirrhosis, severe gastric coronary vein varices, and splenomegaly induced by portal hypertension. (b) Biopsy with the forceps before balloon dilation of the shunt under X-ray (the arrow pointed at the tip of the forceps). (c) Angiography showed that TIPS was performed successfully after the biopsy, with no procedure-related complications. (d) Pathological characteristics of the liver tissues obtained during TIPS highlighted the fibrous septum with disturbance of hepatic lobule (arrow), which meant grade III fibrosis (H&E staining, ×100).

Between January 2009 and December 2012, 452 patients underwent TIPS at the Department of Interventional Therapy, Beijing Shijitan Hospital, Capital Medical University (Beijing, China). Among these patients, 361 patients were enrolled into this study according to the inclusion and exclusion criteria (Figure 1). After hospitalization, a magnetic resonance imaging of the portal vein (MRPV) (Figures 2(a) and 3(a)) was performed on each patient. Laboratory tests, including alanine aminotransferase (ALT), aspartate aminotransferase (AST), and blood ammonia, were recorded for each patient before TIPS. All the patients underwent the TIPS procedure and biopsy. Complete clinical and pathological data were collected retrospectively for those patients.

### 2.2. Procedures of TIPS

The TIPS procedure under general anesthesia was conducted in the Interventional Radiology Center. The Rösch Uchida Transjugular Liver Access Set (Cook, Bloomington, IN, United States) was used. The right internal jugular vein puncture was performed and a 10-F sheath was placed in the vein. After a 5-F multipurpose catheter was used to engage the right hepatic vein, a 10-F curved cannula was delivered into the hepatic vein. A puncture needle in a sheath was advanced into the portal vein through the liver parenchyma and the guide wire was placed into the portal vein through the sheath. A 5-F pigtail catheter was used for angiography and pressure measurement of the portal vein, and an 8 mm or 10 mm diameter angioplasty balloon according to the portal vein was introduced along the guide wire to dilate the shunt. Liver tissues were obtained with biopsy forceps before balloon dilation: biopsy forceps (Minimally Invasive Medical Technology Co., Ltd., Nanjing, China) were inserted through the 10-F curved cannula to the liver parenchyma to obtain the liver tissues (Figures 2(b) and 3(b)). After the biopsy, a covered stent (Bard, Fluency) with a diameter of 8 mm or 10 mm according to the portal vein diameter was implanted to the predilated channel. An additional stent was utilized to extend the shunt if one stent was not enough. The varicose coronary gastric vein was embolized to prevent future gastrointestinal bleeding. The portal vein angiography (Figures 2(c) and 3(c)) and portal pressure measurement were performed after the procedure. The portosystemic pressure gradient (PSG) was measured before and after the shunt creation.

### 2.3. Pathological Information and Patient Grouping

Pathological diagnosis was performed for all the liver tissues that were collected during TIPS, to identify the severity of inflammation (Figure 2(f)) and the presence of fibrosis (Figure 3(d)), which were caused by dilation of the liver parenchyma. The patients were divided into 2 groups according to pathological characteristics: (1) group G0–G2 versus group G3-G4 based on inflammation or (2) group S0–S2 versus group S3-S4 based on fibrosis.

### 2.4. Postoperative Treatment and Observation

All the patients were asked to stay in bed for 8 h after the operation; pressure dressing and sand bag pressing were used for the piercing site area, and the vital signs of the patients were real-time monitored. Intravenous injection of branched chain amino acid (250–500 mL, 1 time/day) and oral administration of lactulose (15–30 mL, 2-3 times/day) were used routinely to prevent HE. Liver protection strategy was taken (bicyclol tablets, 25 mg, p.o., 3 times/day).

Demographic and clinical characteristics of the patients 1 week before and after TIPS were collected. Complications including abdominal cavity hemorrhage, subcapsular hematoma, hepatic failure, infection, bile peritonitis, or pneumothorax were closely observed during the perioperative period.

### 2.5. Follow-Up

The patients were routinely followed up for 24 months. Dietary guidance against HE was given to each patient. Clinical and demographic parameters were compared between these groups. The patients underwent ultrasound examination at 1, 3, 6, 12, and 24 months after TIPS placement. Incidence rates of shunt stenosis and HE after TIPS were calculated.

### 2.6. Statistical Analyses

SPSS 17.0 software was used for statistical analyses. Quantitative data was described as mean ± standard division (SD). Qualitative data was described as frequencies and percentages. Student's *t*-test and chi-square test were used for the comparisons of the quantitative and qualitative data. Multivariate logistic regression analysis was used to assess the risk factors related to the endpoints. The odds ratio (OR) values with 95% confidence intervals (CI) were calculated. A *P* value of <0.05 was considered statistically significant.

## 3. Results

### 3.1. Patient Data

A total of 361 patients were enrolled in the study (Table 1). The mean age was 49.90 ± 11.34 years old. The hepatic function status was evaluated by Child-Pugh classification, dividing the patients into 3 groups: 207, 93, and 61 in classes A, B, and C, respectively. The mean MELD score was 10.60 ± 3.11. The ALT, AST, and blood ammonia before TIPS were 45.87 ± 17.88 U/L, 41.02 ± 14.77 U/L, and 82.70 ± 18.48 *μ*mol/L, respectively. The indications for TIPS included gastroesophageal variceal bleeding in 301 patients, refractory ascites in 43 patients, and gastroesophageal variceal bleeding combined with refractory ascites in 17 patients. The mean PSG before and after TIPS shunt creation was 32.86 ± 2.23 mmHg and 10.03 ± 1.87 mmHg, respectively. The 8 mm stent was used in 298 patients, whereas the 10 mm stent was used in 63 patients. The number of stents utilized was 1 in 270 patients and 2 in 91 patients, respectively.

### 3.2. Pathological Examination Results

As for inflammation grading, 235 (65.1%) of the 361 patients were in grades G1-G2 and 126 (34.9%) patients in G3-G4. As for staging of the liver fibrosis, 120 (33.2%) patients were in S1-S2 and 241 (66.8%) patients in S3-S4. The biopsy during TIPS was successful in all cases (100%), and no procedure-related complications including abdominal cavity hemorrhage, subcapsular hematoma, infection, damage of vein, bile peritonitis, or pneumothorax were observed.

### 3.3. Factors Associated with Shunt Stenosis and HE

Shunt stenosis developed in 40 (11.1%) cases within two years. In univariate analysis, the severity of the liver inflammation, MELD score, PSG before TIPS shunting, and PSG reduction were associated with shunt stenosis. The multivariate logistic regression analysis showed that the risk of shunt stenosis was much higher in patients with more severe inflammation in liver tissue (odds ratio [OR], 2.864; 95% CI: 1.466–5.592; *P* = 0.002) or HE occurrence (OR, 6.266; 95% CI, 3.141–12.501; *P* < 0.001). The risk of shunt stenosis increased about 50% for each 1-point increase in the MELD score (95% CI, 1.298–1.731; *P* < 0.001) (Table 2).

In the present study, HE occurred in 86 (23.8%) patients. In univariate analysis, HE after TIPS was associated with staging of the liver fibrosis, inflammation severity, occurrence of shunt stenosis, MELD score, blood ammonia level, ALT level before TIPS, PSG before TIPS, PSG after TIPS, and PSG reduction. In multivariate logistic regression analysis, the risk of HE was much higher in those with high stage of the liver fibrosis (OR, 2.431; 95% CI, 1.355–4.359; *P* = 0.003) or shunt stenosis occurrence (OR, 6.266; 95% CI, 3.141–12.501; *P* < 0.001). The risk of HE increased about 100% for each 1-point increase in the MELD score (95% CI, 1.711–2.406; *P* < 0.001) (Table 3). The correlation of HE and shunt stenosis was 98.0% (*P* < 0.001).

## 4. Discussion

TIPS is an effective and widely used method of treating complications of portal hypertension induced by cirrhosis; however, two serious complications have limited the wide application of this technology. First is the stenosis of the shunt after TIPS. The application of covered stents has greatly decreased the incidence of stenosis of the shunt; however, this complication still occurs, and the exact mechanisms involved in the development of stenosis are unclear. The other drawback of TIPS is HE. The incidence of postoperative HE is about 25%–45% [8, 9], which severely affects the prognosis and quality of life of the patients.

The present study focuses on examining the risk factors associated with shunt stenosis of TIPS using covered stents. Previous studies suggest that stent thrombosis, bile leakage, and pseudo-intima hyperplasia may be the major causes of stenosis of shunt using bare stent [10–12]. Covered stents have been routinely applied in TIPS procedures and have greatly increased the 1-year patency rate to 90–95% [13]. In our study, increased odds of shunt stenosis were associated with more severe inflammation in liver tissue and a high MELD score. The previous studies revealed that increased levels of TNF-*α* as well as IL-2, IL-6, and IL-10 in serum of patients play important roles in the progress of inflammation of severe hepatitis B-related liver cirrhosis [14, 15]. Meanwhile, recent studies demonstrated that inflammation induced by TNF-*α* and ILs was a key node in stenosis of carotid artery stenting (CAS), which could be the target of treating the stenosis [16, 17]. In our study, stenosis of TIPS was also associated with inflammation. Probably, increased secretion of inflammatory cytokines in cirrhosis of high grade inflammation plays a major role in the process of TIPS shunt stenosis. Anti-inflammation therapy might increase the patency rate of TIPS and would serve as a research direction in the future.

**Figure 4:**
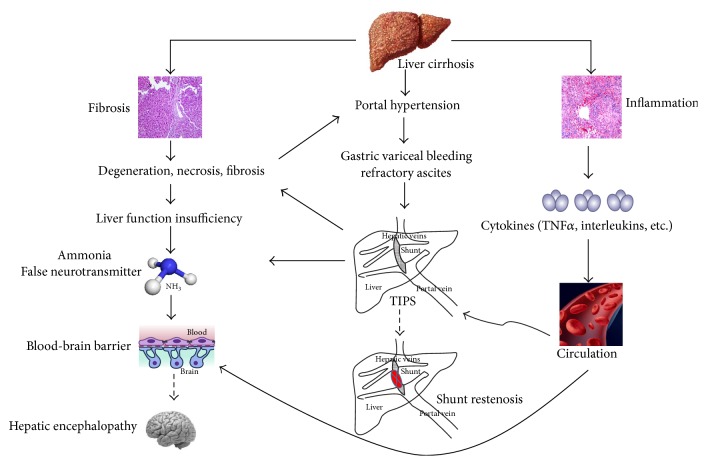
Hypothesis of shunt restenosis and HE after TIPS. Pathological features of the liver tissues predicted the incidence of shunt restenosis and HE. Elevated levels of cytokines in the circulation induced by inflammation play a critical role in restenosis of the stent, which may serve as the target for treatment. Increased blood ammonia and false neurotransmitter result in liver dysfunction induced by fibrosis, which is aggregated by TIPS. In addition, the release of cytokines induced by inflammation can influence the permeability of the blood-brain barrier (BBB), making it easier for the blood ammonia and false neurotransmitter to cross the BBB, leading to the neurodysfunction, and finally promoting the development of HE.

The incidence (23.8%) of post-TIPS HE observed in the present study was similar to previous studies. Merola et al. showed that higher MELD scores, hyponatremia, and higher total bilirubin level were associated with the development of overt HE post-TIPS [18]. Other studies demonstrated that the risk of post-TIPS HE was higher in the patients with increased age, preexisting HE, and higher Child-Pugh score [19, 20]. Although there have been some hypotheses of HE, the underlying molecular mechanisms remain unclear. It has been indicated that blood ammonia in the portal vein bypasses the liver metabolism and then enters the systemic circulation through the shunt after TIPS, and increased blood supply would lead to the dysfunction of the central nervous system and thus HE [21]. In our study, high grade of liver fibrosis and high portal pressure before TIPS were the risk factors of HE. The pathological investigation might explain the results: on the one hand, severe fibrosis of the Disse space and sinusoid capillarization was observed in S3-S4 inflammation group. These changes would restrict the blood flow in portal vein but increase the blood flow in the small collateral vessels. The alteration could reduce the metabolism of blood ammonia in the liver cells and, therefore, increase the level of blood ammonia [22]. On the other hand, the metabolism and detoxification abilities of the liver could be further decreased by more severe inflammation and fibrosis, when the blood flow from the portal vein into the liver was reduced after TIPS. These features could induce the ischemic necrosis of the hepatocytes and liver dysfunction, reduce the metabolism of blood ammonia, and finally increase the incidence of HE occurrence (Figure 4).

This is the study on the correlation between pathological changes of the liver and the clinical features after TIPS, as well as on the risk factors of shunt stenosis after TIPS using covered stents. We found that pathological examination, as the golden standard of a disease, can reveal more information underlying the mechanism of stenosis and HE after TIPS and thus help improve the postoperative survival rate of the patients who undergo TIPS.

There are some limitations in the study. The results did reflect the pathological status before the creation of a shunt; however, the pathological data at the occurrence of shunt stenosis or HE was not acquired. Although the inflammation status of the liver affects the patency of the TIPS shunt, further detection of the inflammatory cytokines should be carried out. All the patients included in this study were suffering from portal hypertension caused by hepatitis B-related cirrhosis, while no patients with hepatitis C or alcoholic cirrhosis were involved.

## 5. Conclusion

The pathological features of the liver tissue obtained during TIPS can help predict the complications including shunt stenosis and HE. Appropriate strategy targeting these critical factors could be taken to improve the postoperative survival rate of the patients who undergo TIPS.

## Figures and Tables

**Table 1:** Demographics and clinical and pathological characteristics of patients undergoing TIPS.

Variable	Overall	Stenosis (*n* = 40)	Nonstenosis (*n* = 321)	*P* value	HE (*n* = 86)	Non-HE (*n* = 275)	*P* value
Age (mean ± SD, years)	49.90 ± 11.34	50.45 ± 10.33	49.83 ± 11.7	0.747	50.37 ± 10.18	49.76 ± 11.69	0.661
MELD score	10.60 ± 3.11	13.05 ± 2.06	10.25 ± 3.05	<0.001	13.52 ± 2.22	9.68 ± 2.77	<0.001
Bilirubin (*μ*mol/L)	33.65 ± 66.83	40.38 ± 58.11	33.25 ± 30.23	<0.001	39.26 ± 50.14	40.15 ± 28.96	<0.001
Creatinine (mg/dL)	0.82 ± 0.45	0.83 ± 0.41	0.81 ± 0.36	0.264	0.89 ± 0.31	0.80 ± 0.44	0.546
INR	1.40 ± 0.32	1.46 ± 0.35	1.39 ± 0.28	0.864	1.45 ± 0.34	1.40 ± 0.29	0.901
Blood ammonia	82.70 ± 18.48	82.95 ± 19.22	82.67 ± 18.41	0.929	86.77 ± 2.05	81.43 ± 17.44	0.035
ALT (U/L)	45.87 ± 17.88	45.38 ± 16.66	45.93 ± 18.05	0.852	49.52 ± 19.98	44.73 ± 17.05	0.047
AST (U/L)	41.02 ± 14.77	43.05 ± 15.71	40.77 ± 14.65	0.358	42.74 ± 14.45	40.49 ± 14.85	0.217
PVP pre-TIPS	40.07 ± 3.25	43.18 ± 3.57	39.81 ± 3.12	<0.001	44.30 ± 2.34	38.75 ± 2.19	<0.001
PVP post-TIPS	11.03 ± 1.87	11.45 ± 2.23	10.98 ± 1.82	0.130	11.85 ± 1.80	10.77 ± 1.82	<0.001
PVP reduction	29.04 ± 3.42	30.73 ± 3.88	28.83 ± 3.30	0.001	32.45 ± 2.81	27.98 ± 2.85	<0.001
Gender							
Male (*n*/%)	237 (65.7)	24 (60.0)	213 (66.4)	0.425	54 (62.8)	183 (66.5)	0.552
Female (*n*/%)	124 (34.3)	16 (40.0)	108 (33.6)		32 (37.2)	92 (33.5)	
Inflammation grading							
G1-G2	235 (65.1)	17 (42.5)	218 (67.9)	0.001	41 (47.4)	194 (70.5)	<0.001
G3-G4	126 (34.9)	23 (57.5)	103 (32.1)		45 (52.3)	81 (29.5)	
Fibrosis staging							
S1-S2	120 (33.2)	8 (20.0)	112 (34.9)	0.074	17 (19.8)	103 (37.5)	0.002
S3-S4	241 (66.8)	32 (80.0)	209 (65.1)		69 (80.2)	172 (62.5)	
HE occurrence					Shunt restenosis occurrence		
Y	86 (23.8)	24 (60.0)	62 (19.3)	<0.001	24 (27.9)	16 (5.8)	<0.001
N	275 (76.2)	16 (40.0)	259 (80.7)		62 (72.1)	259 (94.2)	
Indication of TIPS							
Gastric variceal bleeding	301 (83.4)	35 (87.5)	266 (82.9)	0.865	74 (86.0)	227 (82.5)	0.785
Refractory ascites	43 (11.9)	4 (10.0)	39 (12.1)		8 (9.3)	25 (12.7)	
Both symptoms	17 (4.7)	1 (2.5)	16 (5.0)		4 (4.7)	13 (4.8)	
Child-Pugh classification							
A	207 (57.3)	18 (45.0)	189 (58.9)	0.164	50 (58.7)	157 (57.1)	0.875
B	93 (25.8)	15 (37.5)	78 (24.3)		23 (26.7)	70 (25.5)	
C	61 (16.9)	7 (17.5)	54 (16.8)		13 (15.1)	48 (17.5)	
Number of stents							
1	270 (74.8)	30 (75.0)	240 (74.8)	0.974	61 (70.9)	209 (76.0)	0.345
2	91 (25.2)	10 (25.0)	81 (25.2)		25 (29.1)	66 (24.0)	
Diameter of stent (mm)							
8	298 (82.5)	32 (80.0)	266 (82.9)	0.660	74 (86.0)	224 (81.5)	0.327
10	63 (17.5)	8 (20.0)	55 (17.1)		12 (14.0)	51 (18.5)	

TIPS: transjugular intrahepatic portosystemic shunt; MELD: model for end-stage liver disease; INR: international normalized ratio; ALT: alanine aminotransferase; AST: aspartate aminotransferase; PVP: portal vein pressure; G: grading; S: staging; HE: hepatic encephalopathy.

**Table 2:** Multivariate analysis of the risk factors of stent restenosis.

Index	OR (95% CI)	*P* value
Inflammation grading	2.864 (1.466–5.592)	0.002
MELD score	1.499 (1.298–1.731)	<0.001
HE occurrence	6.266 (3.141–12.501)	<0.001

MELD: model for end-stage liver disease; HE: hepatic encephalopathy.

**Table 3:** Multivariate analysis of the risk factors of HE.

Index	OR (95% CI)	*P* value
Fibrosis staging	2.431 (1.355–4.359)	0.003
MELD score	2.029 (1.711–2.406)	<0.001
Shunt restenosis occurrence	6.266 (3.141–12.501)	<0.001

HE: hepatic encephalopathy; MELD model for end-stage liver disease; PVP: portal vein pressure.
